# Alginate Oligosaccharide Ameliorates D-Galactose-Induced Kidney Aging in Mice through Activation of the Nrf2 Signaling Pathway

**DOI:** 10.1155/2021/6623328

**Published:** 2021-01-09

**Authors:** Hui Pan, Wenjing Feng, Ming Chen, Hong Luan, Yi Hu, Xiaoyue Zheng, Shan Wang, Yongjun Mao

**Affiliations:** ^1^Department of Geriatric Medicine, The Affiliated Hospital of Qingdao University, Qingdao, Shandong 266003, China; ^2^Department of Epidemiology and Health Statistics, The School of Public Health of Qingdao University, Qingdao, Shandong 266021, China; ^3^Department of nephrology, The Affiliated Hospital of Qingdao University, Qingdao, Shandong 266003, China

## Abstract

Aging is an independent risk factor for the development of age-related progressive kidney injury. As a part of the aging process, kidney aging has been indicated to be associated with oxidative stress-induced damage. Ameliorating oxidative damage is therefore considered a promising strategy for delaying kidney aging. Alginate oligosaccharide (AOS) has been reported to have a wide range of biological and pharmacological activities. However, no studies have focused on the role of AOS in delaying the kidney aging process. In this study, we aimed to evaluate the potential effects of AOS on kidney aging and its possible mechanisms. Subcutaneous injection of D-galactose (D-gal) (200 mg·kg^−1^·d^−1^) in C57BL/6J mice for 8 weeks was used to establish the aging model. AOS (200 mg·kg^−1^·d^−1^) was administered via oral gavage for the last four weeks. As a result, AOS inhibited the D-gal-induced upregulation of aging markers and significantly improved the kidney index and kidney function of D-gal-induced mice. In addition, AOS ameliorated the degree of tissue damage and fibrosis in the aging kidney. To further explore the potential mechanisms by which AOS attenuates the kidney aging process, the associated oxidative stress-induced damage was analyzed in depth. The data showed that AOS upregulated the expression of Klotho and decreased malondialdehyde levels by increasing the expression of antioxidant enzymes. Furthermore, our results suggested that AOS activated the nuclear factor erythrogen-2 associated factor 2 (Nrf2) pathway by promoting Nrf2 nuclear translocation in aging mice and upregulated the downstream expression of heme oxygenase-1 (HO-1) and NADPH quinone oxidoreductase 1 (NQO1). In conclusion, the present study demonstrated that AOS is a promising agent for attenuating kidney aging, and the underlying molecular mechanisms are related to the activation of the Nrf2 signaling pathway.

## 1. Introduction

Aging is an extremely complex and unstoppable natural process [1]. As a part of the aging process, kidney aging leads to the inevitable loss of kidney function [2]. According to the free radical aging theory that was first proposed by Hamann, oxidative stress damage caused by reactive oxygen species (ROS) and free radicals plays an important role in the aging process [3]. A noteworthy question is whether oxidative stress is involved in the normal process of kidney aging. Studies have shown that oxidative stress damage is a major contributor to the kidney aging process [4]. Excessive ROS in the aging kidney directly results in oxidative stress, which leads to an imbalance in oxidation-reduction reactions (REDOX), further contributing to the acceleration of kidney aging [5]. The antioxidant defense system has been proven to play a major role in senescence by regulating intracellular redox balance [6]. Therefore, resistance to oxidative stress may be a promising therapeutic strategy for delaying the kidney aging process [7].

Considered a critical transcription factor that regulates intracellular antioxidants, nuclear factor erythrogen-2 associated factor 2 (Nrf2) is indispensable in the induction of various cellular defense mechanisms against oxidative stress [8]. Nrf2 is regulated by kelch-like ECH-related protein 1 (Keap1), which prevents Nrf2 from entering the nucleus. When cells are exposed to oxidative stress, the conformation of Keap1 changes. Nrf2 subsequently separates from Keap1, accumulates and enters the nucleus to bind with antioxidant reaction elements (AREs) and activate the expression of second-stage antioxidant enzymes, such as heme oxygenase-1 (HO-1) and NADPH quinone oxidoreductase 1 (NQO1) [9]. Studies have shown that the expression and activity of Nrf2 decreases in aging mammals, and Nrf2 activation was suggested to be one of the most crucial potential therapeutic targets for aging [10].

D-galactose (D-gal)- induced aging animal models have been widely used to assess the therapeutic effects and mechanisms of natural compounds on oxidative stress damage [11]. Chronic administration of D-gal may cause physiological states similar to those observed during natural aging, such as cell metabolism disorders and injury [12]. Alginate oligosaccharide (AOS) is a degradation product of alginate that has been extensively studied due to its wide range of pharmacological activities and beneficial effects [13, 14]. Previous studies have shown that AOS has a variety of biological activities, such as antioxidant [15], antitumor [16], and immunomodulatory effects [17]. A recent study showed that AOS alleviates morpholine-induced pulmonary hypertension in rats through its antioxidant effects [18]. Based on these studies on the biological activity of AOS, it is reasonable to conclude that AOS may have promising antiaging applications. However, there have been no scientific reports focused on the protective effects and antiaging molecular mechanisms of AOS on D-gal-induced kidney aging in mice.

We hypothesized that AOS delays the kidney aging process by inhibiting oxidative stress by activating the Nrf2 signaling pathway. Therefore, the present study was designed to evaluate the antiaging effects of AOS in D-gal-induced kidney aging in mice and explore its potential mechanism.

## 2. Materials and Methods

### 2.1. Drugs and Reagents

D-gal was purchased from Sigma-Aldrich (St. Louis, USA). AOS was obtained from Qingdao BZ Oligo Biotech Co., Ltd. (Qingdao, China). The commercial kits used to measure blood urea nitrogen (BUN), serum creatinine (Scr), superoxide dismutase (SOD), catalase (CAT), and malondialdehyde (MDA) were supplied by the Nanjing Jiancheng Bioengineering Institute (Nanjing, China). The kit used to extract cytoplasmic and nuclear proteins was provided by Kangwei Century Bioengineering Institute (Beijing, China). The Nrf2, HO-1, NQO1, and GAPDH antibodies were purchased from Elabscience Biotechnology Co., Ltd. (Wuhan, China). The Lamin B rabbit polyclonal antibody was obtained from Santa Cruz Biotechnology, Inc. (Santa Cruz, CA). The p53, p21, Cu/Zn-SOD, Mn-SOD, and CAT antibodies were supplied by Abcam Inc. (Cambridge, UK). All the other chemicals and reagents were purchased from standard commercial suppliers if not otherwise stated.

### 2.2. Animals and Drug Administration

Forty healthy male C57BL/6J mice (8 weeks old, 20 ± 2 g body weight) were purchased from Beijing Weitonglihua Experimental Animal Breeding Co., Ltd (Beijing, China). All mice were housed in the Laboratory Animal Center of the Medical Department of Qingdao University under specific pathogen-free (SPF) conditions (Qingdao, China) and were exposed to a 12-hour light/12-hour dark cycle, with constant temperature (21 ± 2°C) and humidity (60%). All mice could freely access the standard pellet diet and water throughout the experimental period. Animal experimental procedures complied with the “Guide for the Care and Use of Laboratory Animals” published by the US National Institutes of Health and the Public Health Service Policy on Humane Care and Use of Laboratory Animals. The research protocols were approved by the Committee on the Ethics of Animal Experiments of Qingdao University.

After one week of adaptive feeding, the mice were randomly divided into four groups (*n* = 10 each group), including the control group (Control), AOS group (AOS), model group (D-gal), and AOS-treated group (D-gal+AOS). Aging was induced in mice in the D-gal and D-gal+AOS groups through subcutaneous injection of D-gal (200 mg·kg^−1^·d^−1^) for eight consecutive weeks, while mice from the control and AOS groups received a subcutaneous injection of an equivalent dose of sterile water (5 ml·kg^−1^·d^−1^) for eight weeks. Based on the original treatment, beginning at the fifth week, mice in the AOS and D-gal+AOS groups were intragastrically administered AOS (200 mg·kg^−1^·d^−1^), and mice in the control and D-gal groups were administered an equivalent dose of saline (10 ml·kg^−1^·d^−1^) by gavage.

### 2.3. Body Weight Measurement and Kidney Index Analysis

During the experiment, body weight was measured weekly. Twenty-four hours after the last administration, all mice were weighed and anesthetized and then sacrificed humanely. The kidneys of each mouse were carefully dissected, and the weights were recorded. The kidney index was calculated with the following formula: kidney index = kidney weight (mg)/body weight (g).

### 2.4. Histological Analysis

The kidney samples were fixed with 4% paraformaldehyde for 24 hours, embedded in paraffin, and cut into approximately 4 *μ*m thick sections, which were processed and stained with hematoxylin and eosin (H&E), Masson's trichrome, and Sirius red to visualize the kidney architecture and kidney fibrosis. The slices were evaluated in a blinded manner under a light microscope (DP73; Olympus).

### 2.5. Determination of Kidney Function

Serum was separated from whole blood by centrifugation at 3000 rpm for 15 minutes at 4°C and then stored at -80°C for subsequent biological analysis. The levels of BUN and Scr were assessed by an assay kit according to the manufacturer's instructions.

### 2.6. Measurement of Oxidative and Antioxidative Parameters

Many studies have indicated that oxidative stress is involved in the aging process, and the antioxidant system could mediate the REDOX balance in cells and improve the state of aging [6]. The activities of SOD and CAT, as well as the level of MDA, in the supernatant of kidney homogenate were determined by commercially available assay kits. All procedures were performed according to the manufacturer's instructions.

### 2.7. Western Blot Analysis

Kidney tissues were lysed by radioimmunoprecipitation assay (RIPA) lysis buffer (Beyotime, China) containing a mixture of protease and phosphatase inhibitors. Then, the supernatant was collected after centrifugation at 12000 rpm at 4°C for 20 minutes. The protein concentration was determined using a Bradford protein assay (BCA) kit (Boster, China). Samples containing equal amounts of protein were electrophoresed on 10% SDS-PAGE gels and then transferred onto polyvinylidene fluoride (PVDF) membranes (Roche, Switzerland). The membranes were blocked with 5% fresh nonfat milk dissolved in Tris-buffered saline with Tween 20 (TBST) for 1.5 hours at room temperature and then incubated with the following primary antibodies at 4°C overnight: Nrf2, HO-1, NQO1, p53, p21, Cu/Zn-SOD, Mn-SOD, CAT, lamin B, and GAPDH. After being washed with TBST for 30 minutes (3 times, 10 minutes each time), the membranes were incubated with the appropriate horseradish peroxidase-conjugated secondary antibodies (Elabscience, China) at a dilution of 1 : 5000 for 1 hour at room temperature. Subsequently, the membranes were washed in TBST for another 30 minutes, and immunological signals were detected using an enhanced chemiluminescence (ECL) kit (Millipore, USA). The band intensities were quantified by using the ImageJ analysis software. GAPDH was used as an internal control.

### 2.8. Reverse-Transcription Quantitative Polymerase Chain Reaction (RT-qPCR)

Total RNA was extracted from kidney samples using TRIzol reagent (Life Technologies, Carlsbad, CA, USA) under RNase-free conditions. The concentration and purity of the total RNA were determined using an ultraviolet spectrophotometer (Implant, Munich, Germany). The synthesis of cDNA was performed using a Transcriptor First Strand cDNA Synthesis Kit (Roche, Mannheim, Germany) according to the manufacturer's protocol. Real-time quantitative PCR was performed using a LightCycler® 96 system (Roche, Indianapolis, IN, USA). The reaction conditions were as follows: 95°C for 10 min; 95°C for 10 s, 60°C for 10 s, and 72°C for 15 s for forty cycles. The GAPDH gene was used as a reference data normalization. The relative gene expression was calculated using the comparative 2^−△△Ct^ method. The primer sequences (Beijing Ruibo Xingke Biotechnology Co., Ltd.) are listed in Table 1.

### 2.9. Statistical Analysis

All data were obtained from the results of three independent experiments and are expressed as the means ± SEM. All analyses were performed using the SPSS statistical software (SPSS, Chicago, USA), and the differences between groups were determined by one-way analysis of variance (ANOVA) followed by the Student-Newman Keuls (SNK) post hoc test for multiple comparisons. *P* values < 0.05 were considered to be statistically significant.

## 3. Results

### 3.1. Effects of AOS on Body Weight and Kidney Index in Mice

At the beginning of the experiment, there were no differences in the average body weights of the mice in the different groups. As shown in Table 2, the experimental mice showed a significant trend in weight gain; however, compared with those of the control group, the weight and kidney coefficients of mice in the model group were significantly decreased, which was significantly recovered after 4 weeks of AOS treatment.

### 3.2. AOS Improved Kidney Function in D-gal-Induced Aging Model Mice

**Figure 1:**
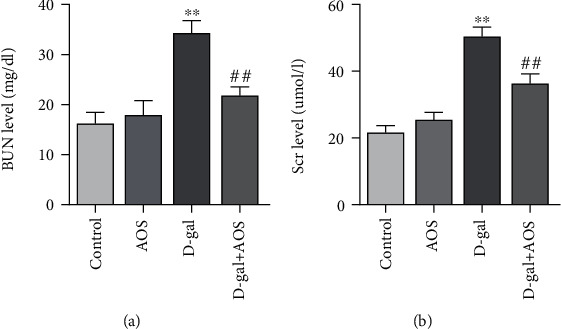
AOS improved kidney function in D-gal-induced aging mice: (a) the level of BUN; (b) the level of Scr. ^∗∗^*P* < 0.01 vs. control group; ^##^*P* < 0.01 vs. model group.

Kidney function was measured to assess the effects of AOS on kidney function in the mice. As shown in Figure 1, the BUN and Scr levels of mice in the model group were significantly higher than those of mice in the control group. However, after AOS supplementation for 4 weeks, the levels of BUN and Scr were lower than those of the model group. These results suggested that AOS alleviated kidney damage induced by D-gal.

### 3.3. Effects of AOS on Kidney Histopathology

**Figure 2:**
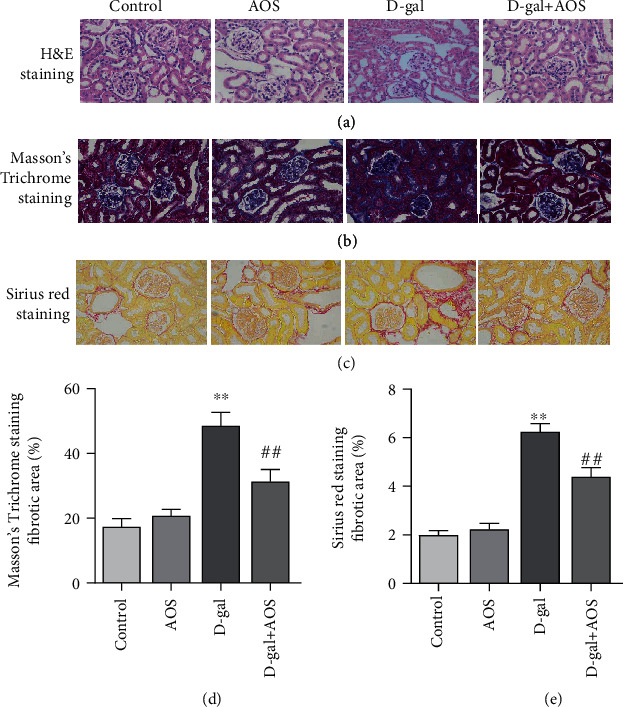
AOS prevented kidney histological alterations in D-gal-induced aging mice. (a) Histomorphological changes were assessed by H&E staining (400x). (b, c) The degree of kidney fibrosis was investigated by Masson's trichrome staining and Sirius red staining (400x). (d) The corresponding quantitative data of (b). (e) The corresponding quantitative data of (c). ^∗∗^*P* < 0.01 vs. control group; ^##^*P* < 0.01 vs. model group.

H&E staining was used to investigate the effects of AOS on the histopathological features of the kidney. As shown in Figure 2(a), in the control group, the kidney presented normal tissue structure, size, and shape in the glomeruli and lacked inflammatory cell proliferation, atrophy, or sclerosis in kidney tubules. Compared with those of the control group, injection of D-gal resulted in glomerular sclerosis and cavity expansion in the damaged kidney with a decrease in the number of normal glomeruli. Sclerosis, atrophy, edema, and vacuolar degeneration in kidney tubules and kidney interstitial fibrosis with inflammatory cell infiltration were also observed in our experiments. However, these pathological changes were significantly attenuated when AOS was administered. To clarify the degree of kidney fibrosis, kidney cross-sections were stained with Masson's trichrome and Sirius red. As shown in Figures 2(b) and 2(c), significant differences in collagen accumulation in the kidney stroma and perivascular areas were observed between the model group and the AOS treatment group. AOS administration obviously decreased the collagen area in D-gal-induced aging kidneys. These results suggested that AOS prevented kidney morphological changes and fibrosis in D-gal-induced aging mice.

### 3.4. AOS Downregulated the Expression of Aging Markers in D-gal-Induced Aging Mice

**Figure 3:**
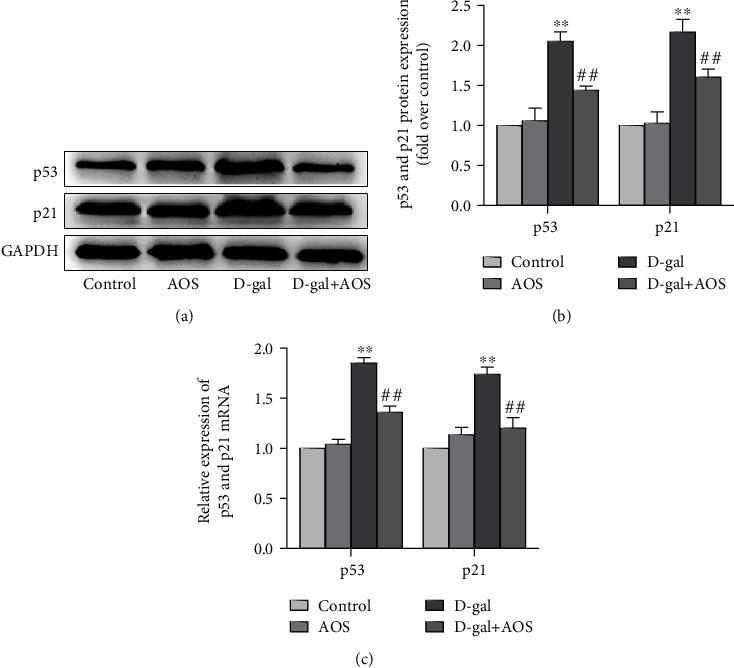
AOS downregulated the expression of aging markers in D-gal-induced aging mice. (a) The protein expression of p53 and p21 was measured by Western blotting. (b) The corresponding quantitative data of (a). (c) The gene expression of p53 and p21 was measured by RT-qPCR. ^∗∗^*P* < 0.01 vs. control group; ^##^*P* < 0.01 vs. model group.

In our study, the expression of the typical senescence markers p53 and p21 in the kidney was measured by Western blotting and RT-qPCR. The results are shown in Figure 3, and compared to that of the control group, there was an obvious increase in the expression of p53 and p21 in D-gal-induced aging mice. However, the observed increase was significantly decreased after AOS administration.

### 3.5. AOS Upregulated the Gene Expression of Klotho in D-gal-Induced Aging Mice

**Figure 4:**
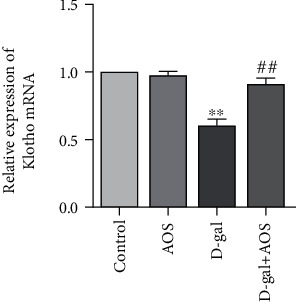
AOS upregulated the expression of Klotho gene in D-gal-induced aging mice. The expression of the antiaging gene Klotho was measured by RT-qPCR. ^∗∗^*P* < 0.01 vs. control group; ^##^*P* < 0.01 vs. model group.

As an antiaging factor, the overexpression of Klotho reduces systemic oxidative stress, thus extending lifespan [19]. To investigate whether AOS regulates the expression of Klotho in the kidney, Klotho mRNA levels were examined by RT-qPCR. As shown in Figure 4, compared with that of the control group, the expression of Klotho was significantly decreased in the model group; however, after AOS was administered for 4 weeks, the mRNA expression of Klotho was increased dramatically. Our results suggested that the Klotho gene may be involved in the mechanisms by which AOS ameliorates D-gal-induced oxidative stress.

### 3.6. AOS Ameliorated Oxidative Stress in D-gal-Induced Aging Mice

**Figure 5:**
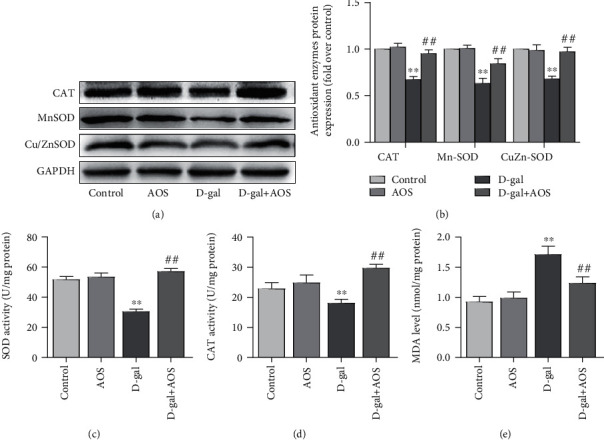
AOS ameliorated oxidative stress in D-gal-induced aging mice. (a) The protein expression of Cu/Zn-SOD, Mn-SOD, and CAT was determined by Western blotting. (b) The corresponding quantitative data of (a). (c) The activity of SOD was measured by commercial SOD assay kits. (d) The activity of CAT was measured by commercial CAT assay kits. (e) The MDA level was evaluated by commercial MDA assay kits. ^∗∗^*P* < 0.01 vs. control group; ^##^*P* < 0.01 vs. model group.

To evaluate the effects of AOS on D-gal-induced oxidative stress in mice, we measured the activities of SOD and CAT, as well as the level of MDA. As shown in Figure 5, compared with those of the control group, the activities and protein expression of SOD and CAT in the D-gal group were significantly decreased. In contrast, the MDA level in the model group was dramatically elevated. As expected, AOS treatment gradually improved these declines and reduced the MDA level. The amelioration of oxidative stress by AOS in D-gal-induced aging mice indicated that AOS may have an effect on the activities of antioxidant enzymes and lipid peroxide levels.

### 3.7. Effects of AOS on the Expression of Nrf2 Signaling Pathway Components in D-gal-Induced Aging Mice

**Figure 6:**
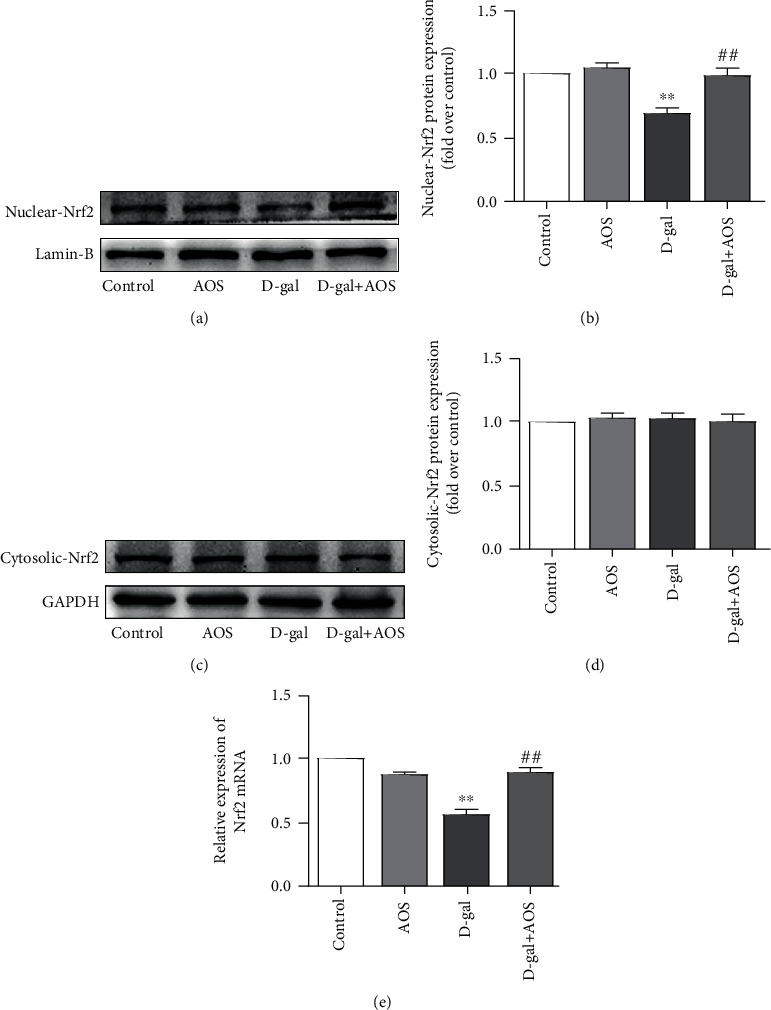
AOS activated the Nrf2 signaling pathway. (a, c) The protein expression of Nrf2 in the nuclear and cytosolic fractions. (b) The corresponding quantitative data of (a). (d) The corresponding quantitative data of (c). (e) The gene expression of Nrf2 was measured by RT-qPCR. ^∗∗^*P* < 0.01 vs. control group; ^##^*P* < 0.01 vs. model group.

Studies have shown that the activity of Nrf2 in aging mammals is significantly reduced, and Nrf2 activation is considered to be a potential target for delaying aging [20]. Nrf2 is a redox-sensitive transcription factor that, once activated, transfers into the nucleus in response to oxidative stress. To further evaluate whether the Nrf2 signaling pathway was involved in AOS-mediated amelioration of D-gal-induced kidney aging, we examined the gene and protein expression of Nrf2 in the nucleus and cytoplasm. The results are described in Figure 6, and the nuclear expression of Nrf2 in the model group was significantly decreased compared with that in the control group. Importantly, the downregulation of the Nrf2 expression was notably reversed after AOS treatment. However, AOS treatment had no significant effects on the expression of Nrf2 in the cytosolic fraction. These findings indicate that Nrf2 is associated with D-gal-induced kidney aging, and the protective effects of AOS on D-gal-induced aging kidney are related to promoting the translocation of Nrf2 protein from the cytoplasm to the nucleus.

**Figure 7:**
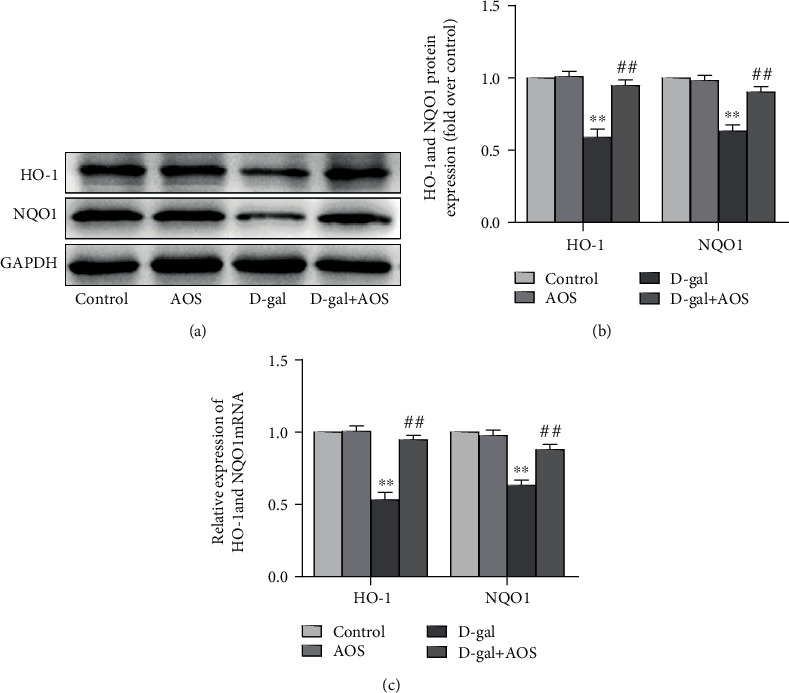
AOS upregulated the expression of HO-1 and NQO1. (a) The protein expression of HO-1 and NQO1 was determined by Western blotting. (b) The corresponding quantitative data of (a). (c) The gene expression of HO-1 and NQO1 was measured by RT-qPCR. ^∗∗^*P* < 0.01 vs. control group; ^##^*P* < 0.01 vs. model group.

To further explore whether AOS was involved in the activation of the Nrf2 signaling pathway, we measured the expression of the downstream genes of Nrf2, the phase II antioxidant enzymes HO-1 and NQO1. Consistent with the above results, the protein and gene expression levels of HO-1 and NQO1 in the model group were significantly lower than those in the control group. As expected, AOS administration enhanced the expression levels of these genes (Figure 7). These results demonstrate that AOS plays an important role in activating the Nrf2 signaling pathway.

## 4. Discussion

Kidney aging is a complex multifactorial process that is associated with cell senescence, oxidative stress, autophagy, and inflammatory responses. In the kidney, the imbalance between the production and elimination of ROS can result in biomolecular damage, thus accelerating the aging process. Accumulating evidence has shown that oxidative stress plays a dominant role in kidney aging by reducing the expression of Nrf2, a critical transcription factor that regulates intracellular antioxidants [21]. The injection of D-gal has been proven to be an effective method for establishing animal models to study aging and test natural antiaging drugs [11]. D-gal-induced kidney aging in C57BL/6J mice was chosen in the current study to investigate the possible antiaging effects of AOS and explore its underlying mechanisms. Our study showed that kidney aging induced by D-gal in mice may be related to oxidative stress damage. AOS treatment has a significant protective effect against this damage, and the mechanisms may be closely related to activation of the Nrf2 signaling pathway.

D-gal, a natural nutrient that is present in the body, may lead to redox disorders when its concentration in cells exceeds the metabolic capacity of the cell. ROS and free radicals accumulate, not only destroying normal biomolecules but also, more importantly, resulting in metabolic disorders in vital organs, such as kidney failure [22, 23]. Significant differences in body weight and kidney histology were observed between mice in the control and D-gal groups. In addition, the expression of the aging-related indicators p53 and p21 increased in the D-gal group. These results are consistent with those of previous studies [24], indicating that the D-gal-induced kidney aging model was successfully established in our study.

With the further study of kidney aging, an increasing number of researchers have realized the interaction between kidney fibrosis and kidney aging. Kidney fibrosis is a pathological lesion associated with the progression of all chronic kidney diseases to end-stage kidney disease. It is characterized by glomerulosclerosis, tubulointerstitial fibrosis, kidney angiosclerosis, and excessive deposition of extracellular matrix [25]. Previous studies have shown that aging is the major contributor closely related to the occurrence and development of kidney fibrosis and interstitial fibrosis increase in the aging kidney [26, 27]. Chung et al. used 6-, 12-, 18-, and 24-month-old SD rats to investigate the changes in kidney fibrosis, and aged kidneys showed increased fibrosis, function decline [28]. In the study of the antiaging effects of Ginsenoside Rg1, results of Masson trichrome staining showed that the level of collagen IV (which is a marker of fibrosis) and the TGF-*β*1 expression (an important factor in promoting the formation of fibrosis) in the kidney was significantly increased in the SAMP8 mice compared with the SAMR1 mice. However, 9 weeks of Rg1 treatment obviously decreased the kidney fibrosis in SAMP8 mice [29]. In this study, we demonstrated intensified interstitial fibrosis in the kidney from D-gal-induced aging mice by Masson's trichrome staining and Sirius red staining and observed significant remission of interstitial fibrosis, glomerular, and kidney angiosclerosis after 4 weeks of AOS treatment. These results suggested that AOS therapy has a protective effect on the development of kidney fibrosis in D-gal-induced aging mice.

It is believed that the occurrence of oxidative stress damage is connected with increased levels of oxidants and the failure of antioxidant mechanisms, which lead to the structural damage of macromolecules, resulting in the corresponding functional decline or loss [30]. Increasing evidence has shown that the excessive production of ROS in biological systems can lead to tissue oxidative damage and affect cell metabolism, especially in the kidney, which is an organ with fast metabolic processes [31]. As the endogenous antioxidant defense system, the main functions of SOD and CAT are to catalyze peroxide redox reactions to hydrogen and reduce lipid peroxidation products, which maintain the balance of ROS in vivo [32]. MDA is one of the most important products of membrane lipid peroxidation, which can exacerbate organ damage [33]. A study on the effects of rhein lysinate on D-gal-induced aging mouse kidneys showed decreased antioxidant enzyme activities, which were associated with impaired kidney function [34]. Consistent with those of previous studies, our findings indicated that AOS exhibited favorable antioxidant effects by enhancing the activities of SOD and CAT and decreasing the level of MDA.

Klotho was first identified by Kuro-o et al. in 1997 as an aging suppressor gene in mice [35]. Studies have shown that Klotho is mainly distributed in distal convoluted tubules of the kidney, the choroid plexus, and parathyroid glands and to a lesser extent in the lung, adipose tissue, and other tissues [36]. Further evidence has shown that Klotho overexpression protects normal cells from oxidative stress and extends life expectancy. Conversely, the downregulation of the Klotho expression accelerated the development of the senescence phenotype [37, 38]. Zeng et al. reported that the glycoprotein isolated from Fupenzi (Rubus chingii Hu.) exerted antiaging and renoprotective properties by improving the Klotho gene expression [39]. The results indicated that the upregulation of Klotho may be a potential strategy to delay renal aging. In our study, AOS increased the gene expression of Klotho in D-gal-induced kidneys, which may be related to the AOS-induced amelioration of kidney aging.

As previously discussed, the Nrf2 pathway is one of the most important signaling pathways in antagonizing oxidative stress injury. Under normal physiological conditions, Nrf2 binds to Keap1 in the cytoplasm and is rapidly degraded by the hydroxylation-ubiquitinated proteasome pathways [40]. When attacked by oxidative stress, the Nrf2/Keap1 complex dissociates, and Nrf2 escapes Keap1-mediated proteasomal degradation and is transferred to the nucleus, where it binds to AREs and induces the transcription of genes encoding antioxidant proteins and phase II detoxification enzymes, such as HO-1 and NQO1 [41, 42]. In recent years, Nrf2 has been considered to be emerging therapeutic target. Studies on animal models with Nrf2 gene knockout in the kidney, liver, brain, and other organs have shown that dysregulated or deficient Nrf2 activity seriously affects the sensitivity of cells to oxidative stress, resulting in some tissues being prone to oxidative stress damage [43]. In the model of ischemia-reperfusion injury, kidney function and the survival rate of Nrf2-knockout mice were significantly worse than those of wild-type mice [44]. In human kidney mesangial cells, high glucose-induced production of ROS inhibited Nrf2 and its downstream genes, ultimately exacerbating oxidative stress. Accordingly, treatment with an Nrf2 activator exerted protective effects against oxidative stress damage in experimental models in vitro and in vivo [45]. Resveratrol and curcumin have been investigated for their ability to attenuate oxidative stress in aging kidneys through the regulation of the Nrf2 signaling pathway [46]. Our further investigations indicated that AOS supplementation potently activated the Nrf2 signaling pathway by increasing the Nrf2 transcription level and facilitated the expression of the Nrf2 downstream target genes HO-1 and NQO1 in aging model mice. These results are in line with those of previous studies [7, 47].

AOS has multiple pharmacological effects; however, in our study, we only explored the antioxidant mechanisms mediated by the Nrf2 signaling pathway in the kidney aging process. Other mechanisms by which AOS protects aging kidneys need to be further explored.

## 5. Conclusion

In summary, the present study confirmed that the Nrf2 signaling pathway was downregulated in the kidney tissue of C57BL/6J mice with D-gal-induced aging. Our investigation demonstrated that AOS significantly alleviated D-gal-induced kidney aging by activating the Nrf2 pathway and enhancing the endogenous antioxidant defense system. These findings indicated that AOS may be a potential agent for delaying kidney aging.

## Figures and Tables

**Table 1:** RT-qPCR primer sequences.

Gene	Primer sequence	Product size (bp)
p53	F: 5′-GCATGAACCGCCGACCTATCC-3′ R: 5′-CCCAGGGCAGGCACAAACAC-3′	187
p21	F: 5′-TCCTGGTGATGTCCGACCTGTTC-3′ R: 5′-CGGCGCAACTGCTCACTGTC-3′	147
Klotho	F: 5′-TGGGTCACTGGGTCAATCTCTGG-3′ R: 5′-GCCTTTCAGCCGCCCTAAAC-3′	143
Nrf2	F: 5′-GTAGATGACCATGAGTCGCTTGCC-3′ R: 5′-CTTGCTCCATGTCCTGCTCTATGC-3′	148
HO-1	F: 5′-ACCGCCTTCCTGCTCAACATTG-3′ R: 5′-CTCTGACGAAGTGACGCCATCTG-3′	104
NQO1	F: 5′-CTCGTAGCAGGATTTGCC-3′ R: 5′-GAAGCCACAGAAACGCA-3′	186
GAPDH	F: 5′-CCTTCCGTGTCCCCACT-3′ R: 5′-GCCTGCTTCACCACCTC-3′	100

**Table 2:** AOS treatment restored the weight and kidney coefficients in D-gal-induced C57BL/6J mice.

Group	Body weight (g)		Kidney weight (mg)	Kidney index (mg/g)
	Initial	Final		
Control	20.55 ± 0.56	28.74 ± 1.30	202.30 ± 8.94	7.04 ± 0.39
AOS	20.68 ± 0.56	29.55 ± 1.73	205.80 ± 7.23	6.99 ± 0.54
D-gal	20.57 ± 0.40	25.85 ± 1.82^∗∗^	153.04 ± 9.12^∗∗^	5.94 ± 0.69^∗∗^
D-gal+AOS	20.67 ± 0.63	28.77 ± 1.84^##^	199.400 ± 9.62^##^	6.96 ± 0.68^##^

^∗∗^ *P* < 0.01 vs. control group; ##*P* < 0.01 vs. model group.

## Data Availability

All data generated or analyzed during this study are included in this published article.
